# Risk of Chronic Obstructive Pulmonary Disease and Receipt of a Breathing Test in 26 States and the District of Columbia, 2017–2018

**DOI:** 10.5888/pcd21.230399

**Published:** 2024-05-09

**Authors:** Kathleen B. Watson, Janet B. Croft, Anne G. Wheaton, Yong Liu, Antonello Punturieri, Lisa Postow, Susan A. Carlson, Kurt J. Greenlund

**Affiliations:** 1 Division of Population Health, National Center for Chronic Disease Prevention and Health Promotion, Centers for Disease Control and Prevention, Atlanta, Georgia; 2 Division of Lung Diseases, National Heart, Lung, and Blood Institute, National Institutes of Health, Bethesda, Maryland

## Abstract

We estimated the prevalence of respiratory symptoms, chronic obstructive pulmonary disease (COPD) risk level, and receipt of a breathing test among adults without reported COPD in 26 states and the District of Columbia by using 2017–2018 Behavioral Risk Factor Surveillance System data. Among adults without reported COPD, the 3 respiratory symptoms indicating COPD (chronic cough, phlegm or mucus production, shortness of breath) were common (each >10%). About 15.0% were at higher COPD risk (based on the number of symptoms, age, and smoking status); 41.4% of adults at higher risk reported receipt of a breathing test. Patient–provider recognition and communication of risk symptoms, appropriate screening, and follow-up are important for early diagnosis and treatment.

**Summary**

**What is already known about this topic?**

More than 14 million Americans have chronic obstructive pulmonary disease (COPD); many more are unaware they have COPD. Untreated, COPD can advance faster to more severe stages of the disease; thus, early diagnosis and treatment are important to reduce death and disability.

**What is added by this report?**

Among adults without reported COPD, the 3 respiratory symptoms that indicate COPD were common (each >10%), and 15.0% were at higher COPD risk; 41.4% of those at higher risk reported receipt of a breathing test.

**What are the implications for public practice?**

Strategies to improve patient awareness and patient–provider communications and promote use of breathing tests (spirometry) in patients at higher risk of COPD are needed to support early screening, diagnosis, and management of COPD.

## Objective

Chronic obstructive pulmonary disease (COPD) is a group of progressive airflow obstruction conditions with limited reversibility. In 2021, 14.2 million US adults reported physician-diagnosed COPD (1); many more adults with lung obstruction are unaware they have COPD (2). Adults at higher risk may not be aware of relevant risk factors and may not recognize symptoms as possibly related to COPD (3). When left untreated, COPD can advance faster to more severe stages of the disease (4); thus, early identification of COPD can improve health and reduce disability and death. Although screening asymptomatic adults is not recommended (5), the Global Initiative for Chronic Obstructive Lung Disease (GOLD) suggests adults with risk factors or symptoms (at higher COPD risk) be directed to subsequent testing for airflow obstruction (6). Estimates of adults at higher COPD risk who received a breathing test (spirometry) are limited. We examined COPD prevalence and, among adults without reported COPD, the prevalence of respiratory symptoms, COPD risk factors, and receipt of breathing test stratified by COPD risk group.

## Methods

We used data from the 2017–2018 Behavioral Risk Factor Surveillance System (BRFSS) (7). Twenty-six states and the District of Columbia administered an optional respiratory health module with questions about 3 respiratory symptoms (chronic cough, phlegm or mucus production, shortness of breath) and receipt of a breathing test. Combined landline and cellular telephone response rates ranged from 35.1% to 64.1% (median, 46.5%). The age-adjusted prevalence of COPD was estimated overall and by subgroup. Among adults without reported COPD, we estimated the age-adjusted prevalence of reported symptoms; the combined number of risk factors and symptoms; the risk for COPD (at higher risk vs not at higher risk); and receipt of a breathing test overall and by subgroup. The 2 risk factors considered were being aged 40 years or older and being a former or current smoker. We determined risk status by using GOLD’s “Could It Be COPD?” questionnaire (8), which asks about the number of risk factors and COPD-related symptoms. We defined higher COPD risk as having 3 or more symptoms or risk factors (combined). We used *t* tests to assess pairwise differences in the prevalence of COPD and COPD risk group by selected characteristics. Prevalence ratios (PRs), estimated from age-adjusted multiple logistic regression models, described the association between risk groups and receiving a breathing test. We used SAS-callable SUDAAN version 11.0.1 (RTI International) to conduct all analyses.

## Results

Information on COPD diagnosis, respiratory symptoms, and receipt of breathing test was reported by 158,741 (82.8%) respondents in 26 states and the District of Columbia. COPD prevalence was highest among women, adults aged 40 years or older, former or current smokers, and adults with current asthma (Table 1). COPD prevalence was higher among non-Hispanic Black and non-Hispanic White adults than among Hispanic adults and increased with decreasing levels of education and urbanicity (Table 1).

**Table 1:** Prevalence^a^ of a Reported Diagnosis of COPD Among All Adults and Prevalence^a^ of Respiratory Symptoms Among Adults Without a Reported Diagnosis of COPD, by Selected Characteristics, Behavioral Risk Factor Surveillance System, 26 States and the District of Columbia, 2017–2018

Characteristic	Prevalence of a reported COPD diagnosis^b^^,^ c (unweighted n = 15,070)	Prevalence of respiratory symptoms among adults without a reported COPD diagnosis^b^ (unweighted n = 143,671)		
		Chronic cough	Sputum production	Shortness of breath
**Total**				
Crude	7.4 (7.2–7.7)	13.7 (13.2–14.1)	12.1 (11.7–12.5)	19.0 (18.5–19.5)
Age-adjusted	6.5 (6.3–6.8)	13.4 (13.0–13.9)	12.0 (11.6–12.5)	18.3 (17.8–18.8)
**Sociodemographic characteristics**				
**Sex**				
Male	5.9 (5.6–6.3)	13.2 (12.6–13.9)	13.1 (12.5–13.8)	14.8 (14.1–15.5)
Female	7.1 (6.7–7.5)	13.6 (12.9–14.2)	11.1 (10.5–11.7)	21.7 (20.9–22.5)
**Age group, y**				
18–39	2.9 (2.6–3.3)	12.2 (11.4–13.0)	11.9 (11.2–12.7)	14.4 (13.6–15.3)
≥40	10.0 (9.6–10.3)	14.5 (14.0–15.1)	12.3 (11.8–12.7)	21.8 (21.2–22.4)
**Race and ethnicity^d^**				
Hispanic	4.7 (3.8–5.8)	13.3 (11.6–15.1)	11.7 (10.2–13.5)	18.9 (17.0–20.9)
Non-Hispanic Black	6.8 (6.0–7.7)	16.0 (14.8–17.4)	14.4 (13.2–15.7)	24.5 (23.1–26.1)
Non-Hispanic White	6.9 (6.6–7.2)	13.2 (12.7–13.7)	12.2 (11.7–12.7)	17.6 (17.0–18.2)
Non-Hispanic Other	5.6 (4.8–6.7)	13.5 (10.9–16.6)	10.0 (8.3–11.9)	14.6 (12.2–17.4)
**Education^e^**				
High school or less	8.8 (8.3–9.3)	16.2 (15.5–17.0)	15.1 (14.3–15.9)	22.7 (21.9–23.6)
Some college	6.5 (6.0–6.9)	13.8 (12.9–14.8)	12.4 (11.6–13.2)	19.1 (18.1–20.2)
College graduate	3.2 (2.9–3.6)	8.9 (8.4–9.5)	7.4 (6.8–7.9)	10.9 (10.4–11.5)
**Urbanicity^e^**				
Large central metro	5.0 (4.5–5.5)	12.4 (11.5–13.4)	10.4 (9.5–11.4)	17.0 (15.9–18.1)
Large fringe metro	5.7 (5.2–6.3)	12.9 (11.9–13.9)	12.4 (11.4–13.4)	17.3 (16.2–18.3)
Medium metro	7.4 (6.8–8.1)	13.5 (12.6–14.4)	12.0 (11.3–12.8)	18.5 (17.5–19.6)
Small metro	7.8 (7.2–8.4)	14.8 (13.4–16.4)	13.2 (12.1–14.4)	20.2 (18.7–21.8)
Micropolitan	8.0 (7.5–8.7)	14.6 (13.6–15.6)	13.5 (12.6–14.5)	20.1 (19.2–21.1)
Rural (noncore)	8.4 (7.7–9.2)	15.7 (13.9–17.8)	15.0 (13.2–17.1)	20.9 (19.8–22.2)
**Other characteristics**				
**Smoking status**				
Former or current smoker	11.2 (10.7–11.7)	17.8 (17.0–18.6)	17.1 (16.3–17.9)	24.2 (23.3–25.2)
Never smoked	3.1 (2.8–3.4)	10.9 (10.3–11.5)	9.0 (8.5–9.5)	14.9 (14.3–15.5)
**Asthma history**				
Current asthma	24.6 (23.2–26.1)	25.3 (23.1–27.6)	23.2 (21.0–25.6)	46.9 (44.3–49.5)
Former history only	11.8 (9.6–14.3)	14.9 (13.0–16.9)	15.2 (13.2–17.5)	21.1 (19.0–23.5)
Never	4.3 (4.1–4.5)	12.3 (11.8–12.8)	10.9 (10.4–11.3)	15.7 (15.2–16.2)
**Receipt of breathing test^f^**				
No	2.3 (2.1–2.6)	11.3 (10.8–11.8)	10.0 (9.5–10.5)	14.1 (13.6–14.6)
Yes	15.7 (15.0–16.5)	19.6 (18.5–20.7)	18.3 (17.2–19.4)	30.9 (29.6–32.2)

Abbreviation: COPD, chronic obstructive pulmonary disease.

a Unweighted n = 158,741. All values are % (95% CI). Estimates for all characteristics, except age group, were standardized to the 2000 US standard population aged ≥18 years by using 5 age groups. During 2017 and 2018, 26 states and the District of Columbia administered the optional module; Tennessee administered the module for both years; therefore, the weight for each respondent in that state was divided by 2.

b Respondents were classified as having self-reported, physician-diagnosed COPD if they responded yes to the question, “Have you ever been told by a doctor or health professional that you have COPD, emphysema, or chronic bronchitis?” Respondents were classified as having a chronic cough if they responded yes to the question, “During the past 3 months, did you have a cough on most days?” Respondents were classified as having sputum production if they responded yes to the question, “During the past 3 months, did you cough up phlegm or mucus on most days?” Respondents were classified as having shortness of breath if they responded yes to the question, “Do you have shortness of breath either when hurrying on level ground or when walking up a slight hill or stairs?”

c Significant differences in the prevalence of COPD were observed for all sex, age group, smoking status, asthma, and receipt of breathing tests pairs.

d Significant differences in the prevalence of COPD were observed between non-Hispanic White and Hispanic groups, between non-Hispanic White and non-Hispanic Other groups, and between non-Hispanic Black and Hispanic groups.

e Significant increasing linear trends in the prevalence of COPD were observed for decreasing education and urbanicity.

f Respondents were classified as receiving a breathing test if they responded yes to the question, “Have you ever been given a breathing test to diagnose breathing problems?”

Among adults without reported COPD, 13.4% (age-adjusted) reported chronic cough, 12.0% reported sputum production, and 18.3% reported shortness of breath (Table 1). Prevalence of reporting each symptom was highest among adults who were aged 40 years or older, were non-Hispanic Black, had a high school diploma or less, were living in rural areas, were former or current smokers, and had current asthma. Among adults without reported COPD, the prevalence of each symptom was higher among those reporting a breathing test (from 18.3% [sputum production] to 30.9% [chronic cough]) than among those reporting no breathing test (from 10.0% [sputum production] to 14.1% [chronic cough]) (Table 1). The prevalence of having a breathing test was higher among adults with COPD (68.1%; 95% CI, 65.0%–71.1%) than without (24.3%; 95% CI 23.8%–24.9%).

Among adults without reported COPD, 23.3% (age-adjusted) reported no symptoms or risk factors, 61.7% reported 1 or 2 symptoms or risk factors, and 15.0% reported 3 or more symptoms and risk factors (at higher COPD risk) (Table 2). Among adults at higher COPD risk, 41.4% reported receiving a breathing test. Overall, adults at higher COPD risk were nearly twice as likely (PR = 1.9; 95% CI,1.8–2.0) to report receiving a test compared with adults not at higher COPD risk (Figure). Women at higher COPD risk (PR = 2.1; 95% CI, 1.9–2.2) were more likely than men at higher COPD risk (PR = 1.7; 95% CI, 1.6–1.8) to report receiving a test; we observed no other subgroup differences.

**Table 2:** Prevalence^a^ of the Number of Respiratory Risk Factors Among Adults Without a Reported Diagnosis of COPD, by Subgroup, Behavioral Risk Factor Surveillance System, 26 States and the District of Columbia, 2017–2018

Subgroup	Combined no. of risk factors and symptoms^b^					
	Not at higher COPD risk (n = 115,344)			At higher COPD risk (n = 27,514)		
	0	1	2	3	4	5
**Total**						
Crude	20.0 (19.5–20.6)	37.6 (37.0–38.3)	26.0 (25.5–26.5)	10.7 (10.3–11.1)	4.2 (4.0–4.4)	1.5 (1.3–1.6)
Age-adjusted	23.3 (22.7–23.8)	37.5 (36.8–38.1)	24.2 (23.7–24.7)	9.8 (9.5–10.2)	3.9 (3.7–4.1)	1.3 (1.2–1.4)
**Age and smoking status^c^**						
Aged <40 y, never smoker	78.3 (77.1–79.5)	14.7 (13.7–15.8)	5.3 (4.7–6.0)	1.7 (1.4–2.1)	—d	—d
Aged ≥40 y, never smoker	—d	73.0 (72.0–73.9)	17.8 (17.1–18.5)	7.0 (6.4–7.7)	2.2 (2.0–2.5)	—d
Aged <40 y, current or former smoker	—d	64.2 (62.2–66.1)	20.7 (19.1–22.5)	10.0 (8.9–11.3)	5.0 (4.1–6.0)	—d
Aged ≥40 y, current or former smoker	—d	^—d^	59.3 (58.2–60.3)	24.6 (23.7–25.6)	10.5 (9.9–11.2)	5.5 (5.1–6.0)

Abbreviation: COPD, chronic obstructive pulmonary disease.

a All values are % (95% CI). Estimates for all characteristics except age were standardized to the 2000 US standard adult population by using the following age groups: 18–24, 25–34, 35–44, 45–64, and ≥65 years.

b Higher COPD risk was based on Global Initiative for Chronic Obstructive Lung Disease’s “Could It Be COPD?” questionnaire (8) and was defined as the sum of risk factors and symptoms ≥3. The questionnaire asks about 3 respiratory symptoms (chronic cough, phlegm or mucus production, shortness of breath) and 2 risk factors (age ≥40 years and current or former smoker).

c The “Could It Be COPD?” questionnaire asks, “Are you older than 40 years?” We used age ≥40 years to correspond with the validation of this questionnaire and because we assumed the survey was administered at least 1 day after the respondent’s birthday, hence older than 40 years. Adults for whom data were missing for smoking status (n = 813) were not including in COPD risk-level subgroups.

d Number of factors is not possible for this subgroup.

**Figure:**
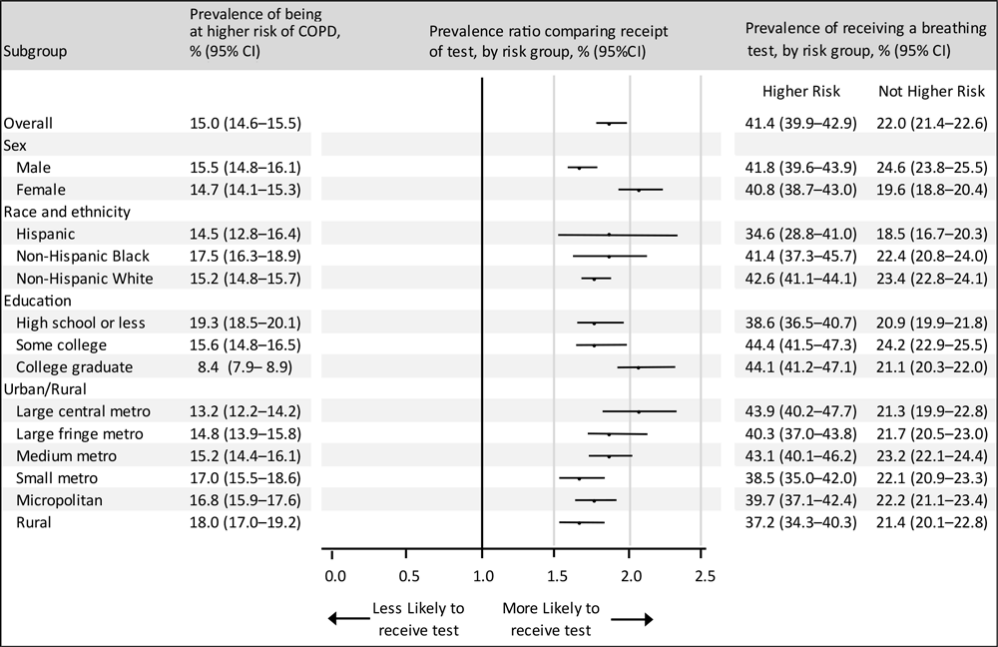
Prevalence of adults at higher COPD risk and prevalence ratios of receipt of a breathing test, by risk status, among adults without a reported COPD diagnosis, by subgroup, Behavioral Risk Factor Surveillance System, 26 states and the District of Columbia, 2017–2018. Estimates were adjusted for age with the inclusion of age group as a covariate in the logistic model (unweighted n = 142,858; 813 responses excluded due to missing data on smoking status); prevalence ratios compare the age-adjusted prevalence of receipt of a breathing test among adults at higher COPD risk versus adults not at higher risk. Higher COPD risk was defined as 3 or more symptoms or risk factors from the GOLD’s “Could It Be COPD?” questionnaire (8). Error bars indicate 95% CIs. “Other” non-Hispanic not included due to heterogeneity of this group. Abbreviations: COPD, chronic obstructive pulmonary disease; GOLD, Global Initiative for Chronic Obstructive Lung Disease.

**Table:** 

Subgroup	Prevalence of being at higher risk of COPD, % (95% CI)	Prevalence ratio comparing receipt of test, by risk group, % (95% CI)	Prevalence of receiving a breathing test, by risk group, % (95% CI)	
			Higher risk	Not higher risk
Overall	15.0 (14.6–15.5)	1.88 (1.80–1.97)	41.4 (39.9–42.9)	22.0 (21.4–22.6)
**Sex**				
Male	15.5 (14.8–16.1)	1.70 (1.59–1.80)	41.8 (39.6–43.9)	24.6 (23.8–25.5)
Female	14.7 (14.1–15.3)	2.09 (1.95–2.23)	40.8 (38.7–43.0)	19.6 (18.8–20.4)
**Race and ethnicity**				
Hispanic	14.5 (12.8–16.4)	1.88 (1.54–2.29)	34.6 (28.8–41.0)	18.5 (16.7–20.3)
Non-Hispanic Black	17.5 (16.3–18.9)	1.85 (1.64–2.09)	41.4 (37.3–45.7)	22.4 (20.8–24.0)
Non-Hispanic White	15.2 (14.8–15.7)	1.82 (1.74–1.90)	42.6 (41.1–44.1)	23.4 (22.8–24.1)
**Education**				
High school or less	19.3 (18.5–20.1)	1.85 (1.72–1.98)	38.6 (36.5–40.7)	20.9 (19.9–21.8)
Some college	15.6 (14.8–16.5)	1.84 (1.69–2.00)	44.4 (41.5–47.3)	24.2 (22.9–25.5)
College graduate	8.4 (7.9– 8.9)	2.09 (1.93–2.26)	44.1 (41.2–47.1)	21.1 (20.3–22.0)
**Urban/rural**				
Large central metro	13.2 (12.2–14.2)	2.06 (1.85–2.30)	43.9 (40.2–47.7)	21.3 (19.9–22.8)
Large fringe metro	14.8 (13.9–15.8)	1.86 (1.68–2.06)	40.3 (37.0–43.8)	21.7 (20.5–23.0)
Medium metro	15.2 (14.4–16.1)	1.86 (1.71–2.02)	43.1 (40.1–46.2)	23.2 (22.1–24.4)
Small metro	17.0 (15.5–18.6)	1.74 (1.57–1.93)	38.5 (35.0–42.0)	22.1 (20.9–23.3)
Micropolitan	16.8 (15.9–17.6)	1.79 (1.65–1.95)	39.7 (37.1–42.4)	22.2 (21.1–23.4)
Rural	18.0 (17.0–19.2)	1.74 (1.57–1.92)	37.2 (34.3–40.3)	21.4 (20.1–22.8)

## Discussion

Among adults without reported COPD, shortness of breath was the most reported symptom (19.0%), followed by chronic cough (13.7%) and sputum production (12.1%). Based on respiratory symptoms, age, and smoking status, 15.0% of adults were at higher COPD risk. Adults at higher COPD risk were twice as likely as adults not at higher COPD risk to report receiving a breathing test, although only 4 in 10 adults at higher COPD risk reported receiving a breathing test.

Studies have consistently shown that COPD symptoms are present in the US population, although prevalence estimates have varied across studies, likely because of differences in study populations and symptom measures (9–11). Reported estimates for wheezing, shortness of breath, and coughing were between 10% and 20% and were dependent on the sample and measure used (9–11). Studies estimating the prevalence of adults at higher COPD risk and how this prevalence is associated with receiving a breathing test are sparser. One study found that 5.1% of adults were at higher COPD risk (12), but their definition of risk differed from ours: their study did not include adults who had symptoms but no smoking history (an estimated 10.9% in our study). Another study reported a similar prevalence for receiving a breathing test (26%), regardless of symptoms or diagnosis (9). Although we found no studies reporting the relationship between risk status and receipt of breathing tests, findings consistently show respiratory symptoms to be present in adults with and without diagnosed COPD.

Early COPD diagnosis leads to treatment and better quality of life. To increase rates of early diagnosis of COPD, it may be important to improve COPD screening through the use of COPD case-finding tools (6). Our study demonstrated that more than half of adults at higher COPD risk had not received a breathing test, a case-finding tool, and may benefit from discussing respiratory symptoms with their health care providers. Improving understanding about the reasons symptomatic adults may be potentially undiagnosed and untreated, such as the cost of testing or incomplete reporting of symptoms by patients (9), can help guide prevention, early diagnosis, treatment, and management strategies (13). Breathing tests are important in diagnosis and guiding treatment decisions for another reason. Respiratory symptoms are common in former and current smokers without airway obstruction (14), yet this group may not respond to COPD therapeutics (15).

This study has limitations. The self-reported data might be subject to recall and social desirability biases. Data included noninstitutionalized adults in 26 states and the District of Columbia; therefore, findings may not be generalizable to the broader US population. Because of differences in the time frame referenced for the symptom-related questions (eg, past 3 months) and the question for receipt of a breathing test (ever), symptoms may or may not have existed before receipt of the breathing test. The presence of respiratory symptoms and risk factors in the absence of diagnosed COPD cannot be interpreted as undiagnosed COPD (11). Finally, when examining the association between COPD risk and receipt of a breathing test, this study did not account for the presence of other respiratory conditions that might warrant a breathing test. For example, 63.6% of adults with and 18.6% without a history of asthma reported a breathing test.

COPD accounts for most deaths from chronic lower respiratory diseases, a leading cause of US deaths (16). Strategies to improve patient awareness and patient–provider communications and to promote the use of breathing tests among patients who are at higher risk for COPD are needed to support the early screening, diagnosis, and management of COPD and its symptoms.
